# Can measurement errors explain variance in the relationship between muscle- and tendon stiffness and range of motion?—a blinded reliability and objectivity study

**DOI:** 10.1007/s00421-025-05814-1

**Published:** 2025-06-11

**Authors:** Konstantin Warneke, Julia Meder, Gerit Plöschberger, Manuel Oraže, Maximilian Zechner, Daniel Jochum, Stanislav D. Siegel, Andreas Konrad

**Affiliations:** 1 Institute of Human Movement Science, Sport and Health, University of Graz, Mozartgasse 14, 8010 Graz, Austria; 2 Department of Human Movement Science and Exercise Physiology, Friedrich Schiller University Jena, Jena, Germany; 3 Institute of Psychology, Leuphana University Lüneburg, Lüneburg, Germany; 4 Viktor Frankl Hochschule, Pädagogische Hochschule Kärnten, Klagenfurt Am Wörthersee, Austria; 5 Department of Health Science and Technology, ETH Zürich, Zurich, Switzerland

**Keywords:** Shear-wave elastography, Myotonometry, Range of motion, Interday reliability, Bland-Altman analysis

## Abstract

**Introduction:**

The relationship between range of motion (ROM) and underlying parameters such as stiffness (ST) remains controversial throughout the literature. Therefore, this study aimed to analyze the potential role of accumulated measurement errors and subjective influences through a comprehensive assessment of both systematic and random errors on the correlation between tissue ST and ROM.

**Methods:**

A total of 75 subjects participated in this double-blinded reliability evaluation. Besides muscle thickness assessments, lower legs’ ST in the calf muscle and Achilles tendon (shear-wave elastography [SWE] and viscoelastic parameters [MyotonPRO], respectively) were correlated with ankle dorsiflexion ROM (knee-to-wall test [KtW]).

**Results:**

Ultrasound image acquisition (i.e., muscle thickness and ST) and myotonometry showed intrasession reliability (ICC = 0.93–0.99 and 0.72–0.99, respectively) depending on the device. Only for MyotonPRO, there were meaningful systematic and random errors only for decrement (SEM = 0.002–10.629; MAE = 0.01–24.84). ROM showed ICC > 0.99, while for all parameters interday reliability declined (ICC = 0.395–0.88). Interrater objectivity showed ICC = 0.61–0.91 for ultrasound analysis and 0.66–0.96 for myotonometry. No agreement (ICC = 0–0.09) between different ST measurements was observed, while relationship between ST and ROM depended on the investigator (*r* = 0.21–0.26 versus *r* = − 0.02–−0.07).

**Discussion:**

While aligned with reliability and objectivity metrics from the literature, our results demonstrate that ST determination is device-dependent, and its relationship with ROM varies by measurement day and investigator. This underlines clinically relevant measurement errors in ST evaluation, calling for advance standardization to improve reliability and objectivity, while measurement errors quantified beyond the ICC must not be neglected in future studies.

## Introduction

In the literature, increasing flexibility and active range of motion (ROM) is described as highly important, considering the discussed influence on joint health (Beselga et al. 2016; Steultjens et al. 2000), athletic performance, and injury prevention (Christopher et al. 2019; de la Motte et al. 2019). Also, several articles have reported that increasing age was associated with decreased ROM parameters, which was assumed a disadvantage as daily activities were negatively affected (Robles-Palazón et al. 2022; Soucie et al. 2011). When explaining variance of ROM, there is an ongoing debate whether on the one hand flexibility is limited by neuronal factors such as pain perception commonly measured via the passive peak torque (Magnusson et al. 1996; Moltubakk et al. 2021; Weppler and Magnusson 2010). On the other hand, stiffer muscles and tendons, or a combination of both (neural and structural), can be responsible for limited ROM (Miyamoto et al. 2018; Reiner et al. 2024).

While the literature provides us with an overflow of evidence, results for the correlation between ROM and muscle stiffness (ST) are controversial. Higher ROM was correlated with lower muscle ST in shear-wave elastography (SWE) (Konrad et al. 2024; Reiner et al. 2024), shear-wave speed (Hirata et al. 2020), strain ratio (Nakagawa et al. 2022), and shear-elastic modulus (Konrad et al. 2024). However, other investigations failed to show associations between ROM and ST (Nakamura et al. 2021) or reported only for specific movements (Reiner et al. 2024). In addition, ST was also quantified with other methods than SWE. Another frequently applied technique to measure ST is performed via dampend oscillation technique, using myotonometry. The controversy can be reviewed for this ST evaluation as well: Some articles present a significant relationship between ST and ROM (Alcaraz-Clariana et al. 2021), while others did not (Chang et al. 2021; Usgu et al. 2023).

How can this discrepancy be explained? When seeking for reasonable interpretations of study results in human research, the precise and adequate measurement becomes paramount, as even small measurement errors can accumulate and result in a clinically relevant conclusion bias (Atkinson and Nevill 1998; Barnhart et al. 2007; Hopkins 2000; Nevill and Atkinson 1997). With regard to precision (refers to the magnitude of random measurement errors when hitting a value) and accuracy (refers to how precisely was the target value hit) (Warneke et al. 2025), it seems remarkable that only a handful of studies did assess reliability for the ultrasound SWE and myotonometry measurements (Agyapong-Badu et al. n.d.; Aird et al. 2012; Bizzini and Mannion 2003; Lee et al. 2021; Agoriwo et al. 2022). Here, the authors collectively focused on relative reliability, i.e., the intraclass correlation coefficients and follow-up calculation such as the standard error of measurement (SEM) and the minimal detectable change (MDC). These, unfortunately, do not account for systematic and random errors with paramount clinical relevance (Lamb 1998), as statistically significant and, following current guidelines, excellently classified ICCs can be accompanied by considerable measurement errors as relative reliability metrics seem to not account for systematic and random errors (Afonso 2025; Warneke et al. 2025). Since the current literature leaves important aspects of measurement error underexplored, we hypothesized that inhomogeneity in correlation results stemmed from unreliable/unobjectively performed data collection (random and/or systematic errors affected the relationship relevantly) neglected. This study performed variance explanation by involving reliability and objectivity quantifications. We hypothesized that differences in intersubject and intersession measurement errors hampered the interpretation of results and could explain the discrepancy observed in the literature.

## Methods

### Preliminary assumptions and experimental approach

**Fig. 1:**
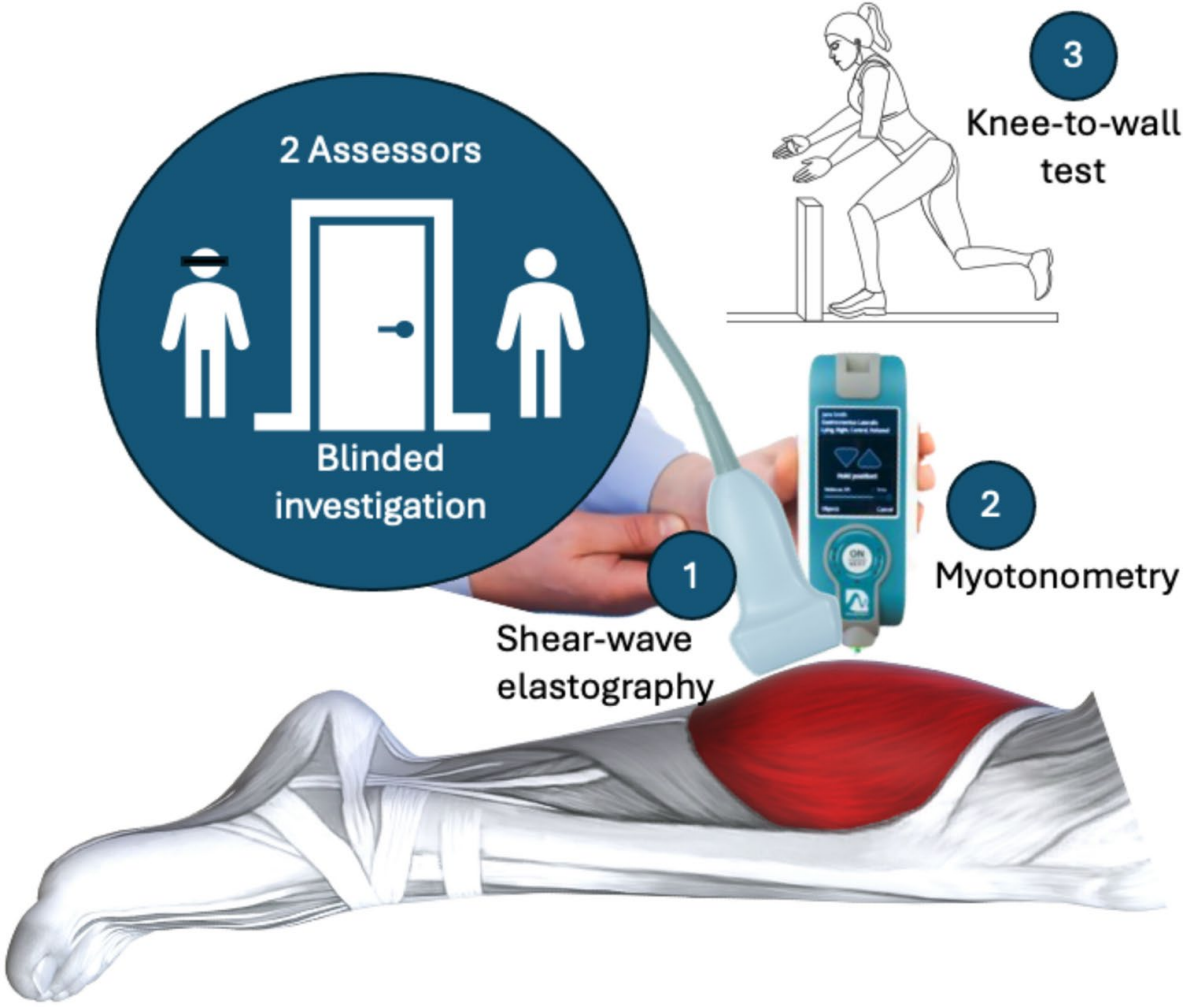
Illustration of the study design, including blinding of participants and the performed measurement

Assuming data collection were performed under reliable conditions, two tests performed twice in a row must produce the same value (intraday reliability). Since significant changes in muscle function and structure are unlikely to occur within a single day, we assumed no day-to-day changes (interday reliability) in the measured parameters. Further, and of relevance for clinical meaningfulness of results, the determination of ST and/or ROM must not depend on the used device, or the investigator (device and investigator objectivity). To explore different sources of measurement errors stemming from intra- and interday differences as well as unclear objectivity, we used ultrasound SWE and myotonometry as two frequently used devices to assess muscle (and tendon) ST. For evaluating ROM, the knee-to-wall test (KtW) was chosen (see Fig. 1). However, also for this test, device and investigator objectivity and intra- and interday reliability were calculated as further sources to explain variance in the correlation between ST and ROM. If a testing protocol produces clinically relevant results, high precision and accuracy of the collected data, expressed in minimal systematic and random error, must be assumed as the preliminary requirement for further calculations. Accordingly, it must be distinguished between statistical and clinical significance when interpreting measurement errors (Willigenburg and Poolman 2023). This, however, would be the case if errors impact the conclusion of the following statistics. Therefore, the relationship between ROM and ST must be consistent across investigators and days. Otherwise, measurement protocol limitations had meaningful influence on the primary rational: The evaluation of the relationship between ROM and the underlying parameter ST.

Beyond, previous results are biased by unreasonable small sample sizes of *n* = 10–40 (Dubois et al. 2015; Lee et al. 2021). Since correlation coefficients and the interpreted relationship between two parameters only stabilized in larger sample sizes (Schönbrodt and Perugini 2013), this limitation was counteracted as well, and 75 participants were recruited. Therefore, statistical power was meaningfully increased by receiving four measurement values per evaluated parameter per participant.

### Participants

A G-Power estimation for correlation studies was performed with a power of 80%, alpha error of 0.05, and effect size of 0.40 (Lee et al. 2021) using G*Power 3.1 software (Heinrich Heine University, Düsseldorf, Germany), and the results showed that the requisite number of participants for this study was 34. To increase statistical power and account for potential dropouts, an overall sample size of *n* = 75 participants (*m*: *n* = 38, age: 28.0 ± 7.3 years ranging from 19 to 40 years, height: 179.5 ± 6.5 cm, mass: 78.9 ± 11.5 kg, *f*: *n* = 37, age: 24.9 ± 3.6 years ranging from 19 to 35 years, height: 168 ± 6.1 cm, mass: 59.7 ± 11.8 kg) were recruited from the university campus out of health- and sport science study programs. Participants were healthy and stated that they did not have any orthopedic, neurological, or cardiorespiratory constraints and were injury-free in the lower extremity since at least 6 months prior to the beginning of the study. All participants were informed about the study protocol and provided written informed consent. The study was conducted in adherence to the Declaration of Helsinki and the procedure was approved by the local ethical review board (No. GZ. 39/145/63 ex 2023/24).

### Testing protocol

After arrival, participants were asked to rest for 5 min in a lying position on the physiotherapy bed, were instructed to the following tests, and provided written informed consent. Time of the day (± 2 h) and room temperature were constant in the test–retest session. The investigators were blinded for the results of the respective other investigator. Additionally, the image evaluation in ultrasound (including muscle architecture and stiffness) was also performed blinded for investigator and measurement day. Between the test–retest session, a maximum of 48 h was ensured.

### Ultrasound investigation

**Fig. 2:**
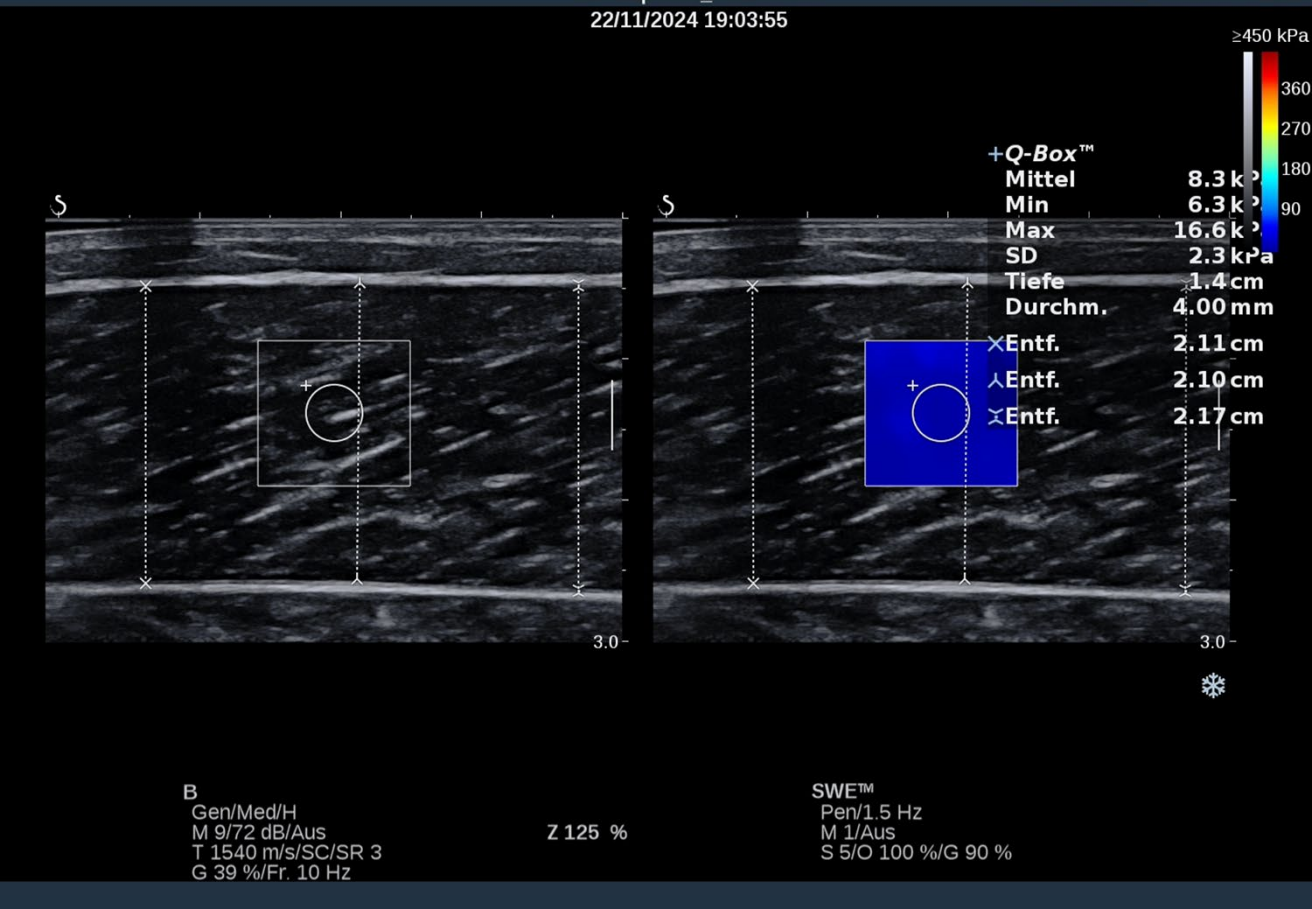
Exemplary shows shear-wave measurement (Q-Box evaluation) and muscle thickness evaluation

Afterward, two investigators marked the position on the medial head of the gastrocnemius where the ultrasound testing was conducted. The position was standardized by determining the position of the muscle tendon junction (distal) and the muscle origin below the knee and taking a distance of 50%. The position was marked by outlining the probe head to ensure that both investigators measured at the same position. Then, the first investigator started the imaging, while the second investigator left the room to be blinded by the results of the first investigator. To collect muscle parameters, the probe (SuperLinear 15–4, 4–15 MHz, Vermont, Tours, France) of the ultrasound device (Aixplorer V12.3, Supersonic Imaging, Aixen-Provence, France) (Brandenburg et al. 2015; Hatta et al. 2015) was positioned on the marked position while the SWE module was turned on. As soon as a stable picture was seen on the device screen, the region of interest was positioned in the mid of the image (see Fig. 2), the fascia superficialis and the deep fascia were parallel, and the image was saved for future evaluation. Muscle thickness was measured using the Aixplorer measurement tool. Three length measurements were taken at three different positions: one on the left, one in the center, and one on the right, from one fascia to the other. The average of these three measurements was calculated to determine the muscle thickness. The muscle stiffness values were evaluated via Q-Box from each image. The procedure was performed twice to determine intrasession reliability for each investigator. As soon as investigator one finished the ultrasound investigation, the assessor changed; investigator one left the room, while investigator two performed testing blinded to the results of the first one. To ensure that the second investigator did not receive information about the results of the first one, as those could affect the second session data collection, a third investigator independently collected data in an excel sheet, to which none of the assessors had access throughout the data collection period. Both investigators were experienced investigators (with up to 12,000 data collections) and have numerous published articles in the field (Konrad et al. 2024; Warneke et al. 2022a, b; Warneke et al. 2024; Warneke et al. 2023a, b, c).

As soon as both investigators finalized their investigation (for muscle thickness and muscle ST in the medial gastrocnemius head), the investigators changed again (the first entered the room again and the second one left), and muscle and tendon ST evaluation using the Myoton Pro started. Of note, the muscle thickness was evaluated as a control parameter for ST measurements, as ultrasound muscle thickness investigations were frequently used in the literature and several studies confirmed reliability of the measurement (Betz et al. 2021; Chiaramonte et al. 2019).

### Muscle and tendon myotonometry

The MyotonPRO (Myoton AS, Tallin, Estland) recorded the damped oscillation of one or several mechanical impulses. For the measurement procedure, the probe was placed perpendicular to the skin overlying the tissue or muscle of interest (Bizzini et al. 2003). With exertion of preload pressure (0.18 N), the device produces a short (0.15 ms) impulse (0.42 N) to the testing area and causes tissue deformation under the testing probe. Together with the released testing probe, the underlying tissue performs damped oscillation, which is recorded by an acceleration-transducer (registration: 385 ms, signal processing: 150 ms, parameter computation: 50 ms). From this signal, the following parameters (Garcia-Bernal 2021): (oscillation) frequency = muscle tone (Hz) described as the natural frequency of the acceleration computed from Fast-Fourier transformation of the signal spectrum, ST (N/m) = the damped natural oscillation response characterizing the resistance to tissue deformation, elasticity = logarithmic decrement as the shape restoration from deformation, relaxation = the time of the muscle recovery process, and creep = gradual tissue elongation under constant stress.

In the systematic review from Lettner et al. (2024), intra-rater reliability for all MyotonPro parameters measured at the gastrocnemius medialis muscle ranged from 0.78 to 0.99. Eight studies measuring the ST at the gastrocnemius medialis were included in the systematic review, but not all of them measured intra-rater reliability for all the parameters. To measure the Achilles tendon ST, a point on the achilles tendon was marked while the markings for the ultrasound were made. The point for the gastrocnemius medialis measurement was set in the middle of the ultrasound marking. The MyotonPRO measurements were conducted in a blinded manner like all the measurements. The data from the MyotonPRO (the five measured parameters that can be read from the MyotonPRO per measurement: *F* = [oscillation] frequency, *S* = stiffness, *D* = elasticity, *R* = relaxation, *C* = creep) were read out loud by the executive investigator and was added to the excel sheet by the third investigator.

### Ankle ROM investigation using the KtW

**Fig. 3:**
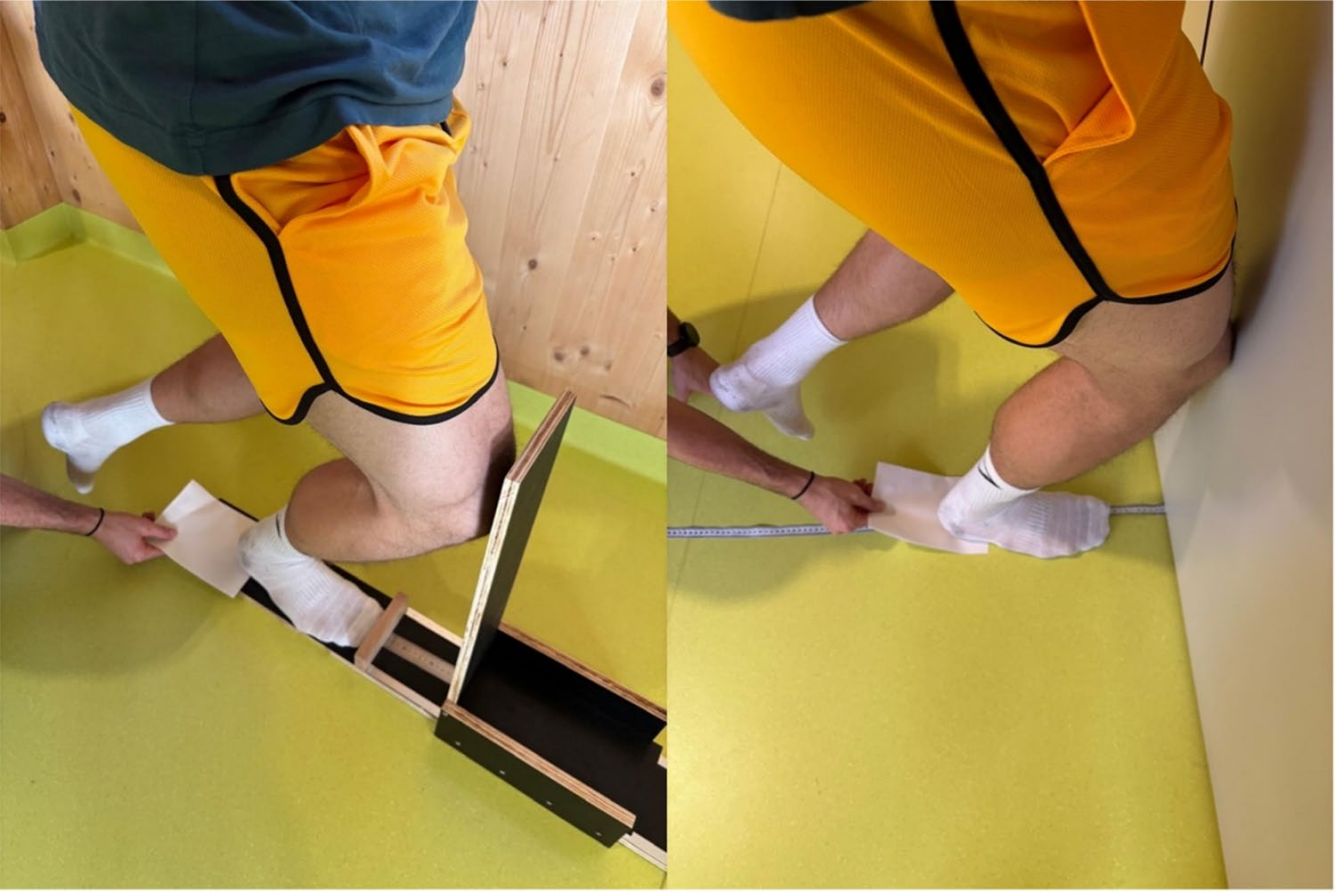
The knee-to-wall test (KtW) with and without device

The KtW is a standard test to assess functional ROM in the ankle and is commonly used to assess calf muscle flexibility (Gould et al. 2024; Warneke et al. 2023a, b, c). To increase standardization of the test and increase precision (moving the foot on a measurement tape allows comparatively unprecise determination, as evaluation is often performed in 0.5 cm steps), the measurement device used by Warneke et al. (Warneke et al. 2022a, b; Warneke et al. 2023a, b, c) seems appropriate to increase precision (see Fig. 3). To date, the device was not evaluated for reliability, validity, and objectivity, yet limiting the practicability in flexibility research and field tests. As ST is one frequently discussed parameter that would influence ROM, the influence of ST on ROM measured via both the common KtW and the device was evaluated in this study. The ROM evaluation was performed as follows: The participant was placed on either the device, or the measurement tape. For testing ROM via the device, the participant was instructed to push the wooden plate forward until their heel lifted. Heel lift was controlled by placing a sheet of paper under the heel, which the investigator constantly pulled on to detect any movement. As soon as the paper could be removed, the test ended, and the distance could be read off with a precision of 1 mm (Fig. 3). The common KtW was performed standing on a measurement tape with the paper sheet placed under the heel. The testing order within the KtW testing was randomized, and the participants were given the opportunity to familiarize with the test procedure before the evaluation started. To determine the upper ankle ROM, the participant was instructed to push the knee forward over the foot, aiming to touch the wall with their knee. If the wall was reached without lifting the heel (the piece of paper remained under the heel), the participant was instructed to move the foot 0.5 cm farther away from the wall. This procedure was repeated until the participant was unable to reach the wall with their knee while the piece of paper remained under their heel. Both tests, again, were performed under blinded conditions, meaning that investigator one was not in the room when investigator two performed the testing and vice versa. The KtW testing order was randomized using lottery. Again, data were inserted into the excel sheet by a third investigator to ensure that testing results were not affected by the results of the respective other.

### Statistical data analysis

To determine inter- and intraday reliability as well as interrater reliability/objectivity, each measurement was conducted twice per investigator and measurement time point. Statistical analysis was performed using JASP (Version 0.18.3 [Intel], Jasp Team [2024], Netherlands). Normal distribution of data was ensured using the Kolmogorov–Smirnov test. Test results were provided as mean (M) and standard deviation (SD). To account for different facets of reliability (intraday, interday, and interrater) and sources of measurement errors, the relative reliability was evaluated using the ICC for agreement (Koo and Li 2016), as follows;

$$\text{ICC}={\text{MS}}_{R}-{\text{MS}}_{E}/\left({\text{MS}}_{R}+\left({\text{MS}}_{C}-{\text{MS}}_{E}\right)/n\right)$$

ICC = Intraclass correlation coefficient,

${\text{MS}}_{C}$ = Mean square for columns,

${\text{MS}}_{E}$ = Mean square for error,

${\text{MS}}_{R}$ = Mean square for rows,

*n* = Number of subjects,

Including the ICC, standard error of measurement (SEM) was calculated using the following formula:

$\text{SEM}=\text{SD}*\sqrt{1-\text{ICC}}$ (Tighe et al. 2010),

where

SEM Standard error of measurement,

SD Standard deviation of the mean difference between trials 1 and 2.

ICC Intraclass correlation coefficient.

In turn, SEM was used to provide the minimal detectable change (MDC), as follows:

$\text{MDC}=\text{SEM}*1.96*\sqrt{2}$, (Seamon et al. 2022).

where

MDC Minimal detectable change

SEM Standard error of measurement

However, to assess whether there was a systematic change (which must be avoided, as no intervention was surrounded by testing), the literature suggests reviewing Bland–Altman (BA) plots and to quantify the mean difference/bias. Since the BA analysis does not provide any significance test for the systematic bias, the mean difference statistical significance can be evaluated using a paired sample *t*-test (Atkinson and Nevill 2000, 1998; Hopkins 2000). In addition, for data interpretation in clinical and practical context, it is important to report the random error/noise (Hopkins 2000), which can be visualized using the BA plots with the respective limits of agreement (LoA), while quantification was suggested using the mean absolute error (MAE) (Willmott and Matsuura 2005; 2006)

$$\text{MAE}=\frac{1}{n}*\sum_{i=1}^{n}\left|{x}_{i}-{y}_{i}\right|$$

where

*n*: Number of data points.
*i*: Index for each (paired) data point.
*x*_*i*_: *i*-th data point in variable *x.*
*y*_*i*_: *i*-th data point in variable *y.*

which can be expressed in percentage using the mean absolute percentage error (MAPE), as follows:

$$\text{MAPE}=\frac{1}{n}*\sum_{i=1}^{n}\left|\frac{{x}_{i}-{y}_{i}}{{x}_{i}}\right|*100$$

where

*n*: Number of data points.
*i*: Index for each (paired) data point.
*x*_*i*_: *i*-th data point in variable *x.*
*y*_*i*_: *i*-th data point in variable y.

Relative reliability metrics were classified using guidelines provided by Koo and Li (2016).

The relationship between ST in the gastrocnemius and Achilles tendon on ROM was evaluated for both the KtW device and in its common way without a device. The assumption that both the Myoton and the SWE would measure ST appropriately was explored by performing a BA analysis with systematic and secondary variance quantification, while the relevance of measuring ST via the Myoton or the SWE on the ROM relationship was investigated by performing Pearson’s correlation between parameters. Inference statistics adhered to an *α*-level of 0.05.

## Results

### Intrasession reliability (day 1)

Ultrasound muscle property investigation provided ICCs between 0.97 and 0.99 for analyzing the muscle thickness and performing the SWE. In accordance with Koo and Li (2016), relative reliability can be classified excellent. The calculated SEM caused errors of 0.003 (both investigators) surrounding mean values of 1.85–1.90 cm for muscle thickness and SEMs of 0.056 and 0.068 for means of 10.98 and 11.30 kPa for muscle ST. In all cases, the MDC (0.008 and 0.009 for muscle thickness and 0.155–0.187) surpassed the systematic bias, which was in none of the cases statistically significant, supporting the classification of excellent reliability. Secondary variance quantification resulted in an MAE of 0.03 and 0.04 for muscle thickness and 0.48 and 0.54 for SWE-based muscle ST evaluation, which corresponds to an MAPE of up to 5%. Although providing ICCs classified as good to excellent (ICC = 0.73–0.99), the myotonometry assessment was accompanied with a statistically significant mean bias for the evaluation of the gastrocnemius medialis and the Achilles tendon in four cases and MAPEs partially surpassing the 10% MAPE with maximum errors of up to 52.73% (parameter “decrement”) (see Table 1).

**Table 1:** Detailed reliability statistics for intraday reliability at day 1, including descriptives (M ± SD) for both tests and investigators, the standard error of measurement (SEM), the minimal detectable change (MDC), as well as the systematic bias with inference statistics, while the mean absolute error (MAE), the mean absolute percentage error (MAPE), and the maximal percentage error (MPE) were used to quantify secondary variance

Parameter	Value1	Value2	ICC (95% CI)	SEM	MDC	Systematic bias	MAE	MAPE (%)	MPE (%)
MTh GM I1	1.89 ± 0.30	1.90 ± 0.31	0.983 (0.97–0.99)	0.003	0.009	0.003 (0.685)	0.04	2.22	12.5
MTh GM I2	1.86 ± 0.30	1.85 ± 0.30	0.985 (0.98–0.99)	0.003	0.008	0.006 (0.313)	0.03	1.84	11.96
ST GM I1	10.91 ± 2.81	10.98 ± 2.90	0.969 (0.95–0.98)	0.068	0.187	0.073 (0.373)	0.54	4.99	17.92
ST GM I2	11.22 ± 2.61	11.30 ± 2.63	0.973 (0.96–0.98)	0.056	0.155	0.072 (0.306)	0.48	4.25	12.64
AT Myo F I1	31.28 ± 2.69	31.06 ± 2.64	0.904 (0.85–0.94)	0.215	0.597	0.225 (0.098)	0.98	3.19	8.87
AT Myo F I2	31.00 ± 2.83	30.84 ± 2.70	0.867 (0.80–0.91)	0.280	0.775	0.161 (0.331)	1.084	3.5	12.61
AT Myo S I1	782.67 ± 96.43	784.23 ± 97.75	0.948 (0.92–0.97)	3.786	10.494	1.560 (0.669)	23.48	3.02	10.87
AT Myo S I2	800.32 ± 95.24	790.24 ± 115.15	0.794 (0.69–0.87)	10.629	29.463	10.080 (0.202)	0.106	3.34	13.82
AT Myo D I1	0.91 ± 0.21	0.87 ± 0.20	0.84 (0.76–0.90)	0.025	0.069	0.035 (0.009)	0.088	10.96	52.73
AT Myo D I2	0.89 ± 0.20	0.89 ± 0.21	0.788 (0.68–0.86)	0.035	0.096	0.006 (0.713)	0.106	12.21	43.66
AT Myo R I1	6.48 ± 0.85	6.51 ± 0.86	0.942 (0.91–0.96)	0.036	0.101	0.024 (0.480)	0.21	3.28	13.72
AT Myo R I2	6.34 ± 0.81	6.37 ± 0.85	0.925 (0.88–0.95)	0.042	0.117	0.033 (0.373)	0.22	3.4	15.79
AT Myo C I1	0.43 ± 0.05	0.44 ± 0.06	0.728 (0.60–0.82)	0.006	0.018	0.004 (0.430)	0.017	3.73	42.25
AT Myo C I2	0.42 ± 0.05	0.43 ± 0.05	0.908 (0.86–0.94)	0.003	0.008	0.002 (0.359)	0.01	3.24	15.79
GM Myo F I1	16.30 ± 2.23	16.38 ± 2.23	0.988 (0.98–0.99)	0.021	0.059	0.089 (0.03)*	0.276	1.7	7.38
GM Myo F I2	16.61 ± 2.19	16.59 ± 2.39	0.943 (0.91–0.96)	0.078	0.217	0.021 (0.812)	0.464	2.77	24.81
GM Myo S I1	285.69 ± 54.50	287.69 ± 56.10	0.967 (0.95–0.98)	1.041	2.886	2.00 (0.226)	8.1	3.07	47.98
GM Myo S I2	293.92 ± 54.96	294.48 ± 56.88	0.966 (0.95–0.98)	1.321	3.662	0.560 (0.741)	10.13	3.46	13.23
GM Myo D I1	1.04 ± 0.15	1.04 ± 0.16	0.896 (0.84–0.93)	0.011	0.031	0.002 (0.782)	0.0489	4.72	25.56
GM Myo D I2	1.06 ± 0.18	1.05 ± 0.17	0.931 (0.89–0.96)	0.009	0.025	0.007 (0.341)	0.0492	4.75	17.04
GM Myo R I1	18.18 ± 2.88	17.98 ± 2.83	0.986 (0.98–0.99)	0.029	0.082	0.195 (< 0.001)	0.352	1.99	12.33
GM Myo R I2	17.77 ± 2.78	17.75 ± 2.86	0.976 (0.96–0.99)	0.044	0.123	0.016 (0.823)	0.405	2.45	23.89
GM Myo C I1	1.11 ± 0.18	1.09 ± 0.16	0.814 (0.72–0.88)	0.010	0.028	0.025 (0.046)	0.0336	3.32	42.03
GM Myo C I2	1.08 ± 0.16	1.08 ± 0.16	0.967 (0.95–0.98)	0.003	0.010	0.0004 (0.933)	0.0268	2.61	22.22
ktW norm I1	13.07 ± 3.19	13.13 ± 3.22	0.997 (0.99–1)	0.005	0.014	0.067 (0.032)	0.133	0.99	6.67
ktW norm I2	13.07 ± 3.35	13.10 ± 3.37	0.995 (0.99–1)	0.010	0.029	0.003 (0.388)	0.2067	1.78	8.33
ktW device I1	12.44 ± 3.56	12.37 ± 3.58	0.994 (0.99–1)	0.016	0.044	0.061 (0.182)	0.288	2.64	16.13
ktW device I2	12.29 ± 3.56	12.31 ± 3.62	0.992 (0.99–1)	0.022	0.061	0.025 (0.636)	0.348	3.2	12.5

MTh = muscle thickness; ST = stiffness; GM = gastrocnemius medialis (MTh GM and ST GM were measured with ultrasound SWE); AT = Achilles tendon; Myo = Myoton; (Myoton parameter: F = (oscillation) frequency, S = stiffness, D = elasticity, R = relaxation, C = creep); KtW norm = Standard Knee-to-wall test; KtW device = Knee-to-wall test with device; I1 = Investigator 1; I2 = Investigator 2

Irrespective of using the device or not, the KtW produced ICCs > 0.99 with only one test (without the device) showing systematic errors for investigator 1 in one session. However, the systematic error was higher using the test device when testing was performed by investigator 1 (systematic bias: 0.06, MDC = 0.04). The MAPE ranged between 0.99 and 3.2%.

### Intrasession reliability (day 2)

On day 2, the relative intraday reliability of the ultrasound investigations (muscle thickness and SWE) was very similar to day 1 with ICC = 0.93–0.99: SEM and MDC for muscle thickness investigation of 0.002 and 0.003, and 0.006 and 0.009, respectively, and 0.057 and 0.112 as well as 0.159 and 0.311 for SEM and MDC for SWE ST determination, respectively. MAEs and MAPEs were comparable to those from day 1 (see Table 2).

**Table 2:** Detailed reliability statistics for intraday reliability at day 2, including descriptives (*M* ± SD) for both tests and investigators, the standard error of measurement (SEM), the minimal detectable change (MDC), as well as the systematic bias with inference statistics, while the mean absolute error (MAE), the mean absolute percentage error (MAPE), and the maximal percentage error (MPE) were used to quantify secondary variance

Parameter	Value1	Value2	ICC (95% CI)	SEM	MDC	Systematic bias	MAE	MAPE (%)	MPE (%)
MTh GM I1	1.88 ± 0.32	1.87 ± 0.32	0.990 (0.98–0.99)	0.002	0.006	0.004 (0.483)	0.032	1.775	11.39
MTh GM I2	1.83 ± 0.31	1.83 ± 0.31	0.983 (0.97–0.99)	0.003	0.009	0.003 (0.666)	0.035	1.89	14.45
ST GM I1	10.91 ± 2.95	10.98 ± 2.89	0.974 (0.96–0.98)	0.057	0.159	0.061 (0.432)	0.504	4.47	12.76
ST GM I2	10.39 ± 2.16	10.51 ± 2.17	0.929 (0.9–0.96)	0.112	0.311	0.121 (0.201)	0.596	5.62	21.48
AT Myo F I1	30.47 ± 2.47	30.69 ± 2.82	0.821 (0.73–0.88)	0.341	0.946	0.219 (0.236)	1.14	3.75	25.22
AT Myo F I2	30.51 ± 2.87	30.49 ± 2.75	0.886 (0.83–0.93)	0.244	0.675	0.023 (0.885)	1.02	3.38	12.89
AT Myo S I1	771.8 ± −82.94	780.13 ± 81.24	0.901 (0.85–0.94)	5.491	15.220	8.253 (0.054)	24.68	3.16	25.28
AT Myo S I2	778.51 ± 93.94	784.65 ± 93.33	0.944 (0.91–0.97)	4.157	11.521	6.147 (0.092)	24.84	3.19	10.73
AT Myo D I1	0.89 ± 0.21	0.89 ± 0.20	0.770 (0.66–0.85)	0.031	0.087	0.004 (0.794)	0.09	9.98	52.7
AT Myo D I2	0.91 ± 0.20	0.89 ± 0.19	0.724 (0.60–0.82)	0.041	0.114	0.019 (0.244)	0.11	12.56	54.38
AT Myo R I1	6.57 ± 0.77	6.47 ± 0.75	0.902 (0.85–0.94)	0.050	0.140	0.097 (0.014)	0.228	3.56	25.76
AT Myo R I2	6.48 ± 0.86	6.45 ± 0.88	0.924 (0.88–0.95)	0.045	0.126	0.028 (0.478)	0.23	3.52	18.42
AT Myo C I1	0.44 ± 0.05	0.43 ± 0.05	0.915 (0.87–0.95)	0.003	0.008	0.006 (0.011)	0.01	3.18	17.78
AT Myo C I2	0.43 ± 0.05	0.43 ± 0.05	0.912 (0.87–0.94)	0.003	0.008	0.001 (0.549)	0.014	3.14	20
GM Myo F I1	16.64 ± 2.38	16.70 ± 2.27	0.954 (0.93–0.97)	0.057	0.158	0.061 (0.455)	0.376	2.17	17.52
GM Myo F I2	16.60 ± 2.36	16.54 ± 2.22	0.966 (0.95–0.98)	0.044	0.123	0.060 (0.390)	0.34	2.05	19.37
GM Myo S I1	291.20 ± 55.86	291.24 ± 54.01	0.969 (0.95–0.98)	1.091	3.023	0.040 (0.980)	8.76	3.07	19.42
GM Myo S I2	292.49 ± 53.21	298.25 ± 70.05	0.845 (0.77–0.90)	2.977	8.252	5.760 (0.153)	10.69	2.93	41.22
GM Myo D I1	1.02 ± 0.14	1.03 ± 0.14	0.922 (0.88–0.95)	0.009	0.024	0.004 (0.527)	0.043	4.34	14.68
GM Myo D I2	1.03 ± 0.14	1.03 ± 0.15	0.910 (0.86–0.94)	0.010	0.028	0.002 (0.743)	0.048	4.58	16.5
GM Myo R I1	17.83 ± 2.94	17.83 ± 2.94	0.978 (0.97–0.99)	0.045	0.124	0.004 (0.955)	0.428	2.47	18.18
GM Myo R I2	17.87 ± 2.89	17.77 ± 2.83	0.988 (0.98–0.99)	0.026	0.072	0.095 (0.064)	0.33	1.84	5.94
GM Myo C I1	1.08 ± 0.16	1.08 ± 0.17	0.961 (0.94–0.98)	0.004	0.011	1.333 * 10^–4^ (0.980)	0.028	2.62	25.49
GM Myo C I2	1.09 ± 0.16	1.08 ± 0.16	0.983 (0.97–0.99)	0.002	0.005	0.009 (0.011)	0.021	1.89	10.53
ktW norm I1	13.16 ± 3.38	13.22 ± 3.40	0.997 (0.995–1)	0.006	0.017	0.064 (0.050)	0.163	1.39	8.33
ktW norm I2	13.12 ± 3.45	13.16 ± 3.48	0.995 (0.99–1)	0.009	0.026	0.040 (0.321)	0.187	1.37	10.71
ktW device I1	12.27 ± 3.64	12.29 ± 3.63	0.992 (0.99–1)	0.020	0.056	0.020 (0.701)	0.321	2.91	16.67
ktW device I2	12.46 ± 3.44	12.52 ± 3.50	0.993 (0.99–1)	0.019	0.052	0.060 (0.220)	0.319	2.77	13.04

MTh = muscle thickness; ST = stiffness; GM = gastrocnemius medialis (MTh GM and ST GM were measured with ultrasound SWE); AT = Achilles tendon; Myo = Myoton; (Myoton parameter: *F* = (oscillation) frequency, *S* = stiffness, *D* = elasticity, *R* = relaxation, *C* = creep); ktW norm = Standard knee-to-wall test; ktW device = Knee-to-wall test with device; I1 = Investigator 1; I2 = Investigator 2

As for day 1, relative reliability of myotonometry was worse compared to SWE investigation with ICCs ranging from 0.72 to 0.99 for muscle and Achilles tendon evaluation. In three parameters, there was a significant systematic error, surpassing the MDC in one case. Also on day 2, especially the parameter “decrement” via the MyotonPRO showed MAPEs surpassing 10% with an MPE of over 52% and one time of 41%. The ktW again showed excellent relative reliability (ICC > 0.99) with MAPEs from 1.37 to 2.91% and an MPE of 16.67%. The results are presented in detail in Table 2.

### Interday reliability (day 1 to day 2)

When reviewing the original values (*M* ± SD), ultrasound results (muscle thickness and SWE) suggest reliable measurements, using the mean of test 1 and test 2 from each testing day as the baseline. However, relative reliability decreased and partly did not reach sufficient repeatability between the days (ICC = 0.43–0.89). Accordingly, for example, for SWE muscle ST investigation, there was an SEM for investigator 2 of 1.10 kPa surrounding a mean of 10.45 kPa or 11.26 kPa, and the MAPE reached ≥ 20%. For the same parameter, there was a significant systematic bias that did not surpass the MDC.

For interday, also myotonometry reliability decreased to ICCs ranging from 0.40 to 0.88, while secondary variance was similar compared to the intraday investigations with, again, peaking for “decrement” with 14.72% and 15.98%. One parameter of the Achilles tendon evaluation with MyotonPRO showed a statistically significant systematic bias, not surpassing the MDC. The only test consistently reaching relative reliability classified excellent is the KtW with and without the device. However, also for both tests, the MAPE showed values between 5.25 and 8.19% (Table 3).

**Table 3:** Detailed reliability statistics for interday reliability, including descriptives (*M* ± SD) for both tests and investigators, the standard error of measurement (SEM), the minimal detectable change (MDC), as well as the systematic bias with inference statistics, while the mean absolute error (MAE), the mean absolute percentage error (MAPE), and the maximal percentage error (MPE) were used to quantify secondary variance

Parameter	Value1	Value2	ICC (95% CI)	SEM	MDC	Systematic bias	MAE	MAPE (%)	MPE (%)
MTh GM	1.90 ± 0.31	1.87 ± 0.32	0.894 (0.84–0.93)	0.024	0.065	0.022 (0.190)	0.102	5.77	29.06
MTh GM I2	1.85 ± 0.30	1.83 ± 0.31	0.856 (0.78–0.91)	0.031	0.087	0.021 (0.268)	0.1796	7.64	71.25
ST GM I1	10.94 ± 2.82	10.94 ± 2.90	0.564 (0.39–0.70)	0.983	2.724	0.046 (0.496)	2.104	19.64	76.11
ST GM I2	11.26 ± 2.60	10.45 ± 2.13	0.429 (0.23–0.60)	1.107	3.070	0.810 (0.007)	2.073	20.92	85.61
AT Myo F I1	31.17 ± 2.60	30.58 ± 2.53	0.660 (0.51–0.77)	0.652	1.806	0.589 (0.018)	1.5807	5.33	33.59
AT Myo F I2	30.92 ± 2.67	30.50 ± 2.73	0.711 (0.58–0.81)	0.575	1.593	0.424 (0.078)	1.512	5.11	43.25
AT Myo S I1	783.45 ± 95.81	776.01 ± 80.04	0.692 (0.55–0.79)	19.391	53.750	7.440 (0.355)	49.413	6.56	47.77
AT Myo S I2	795.28 ± 100.07	781.58 ± 92.33	0.692 (0.47–0.75)	23.632	65.505	13.700 (0.157)	60.22	8.15	63.8
AT Myo D I1	0.89 ± 0.19	0.89 ± 0.20	0.407 (0.20–0.58)	0.075	0.207	0.002 (0.933)	0.137	14.72	86.03
AT Myo D I2	0.89 ± 0.19	0.90 ± 0.18	0.395 (0.19–0.57)	0.080	0.222	0.014 (0.545)	0.145	15.98	68.29
AT Myo R I1	6.50 ± 0.84	6.52 ± 0.74	0.662 (0.51–0.77)	0.188	0.522	0.026 (0.075)	0.458	6.87	36.2
AT Myo R I2	6.35 ± 0.82	6.43 ± 0.91	0.616 (0.45–0.74)	0.228	0.632	0.0112 (0.189)	0.52	7.73	41.01
AT Myo C I1	0.43 ± 0.05	0.44 ± 0.04	0.600 (0.43–0.73)	0.013	0.036	0.0007 (0.893)	0.029	6.54	36.59
AT Myo C I2	0.43 ± 0.05	0.43 ± 0.05	0.611 (0.45–0.74)	0.013	0.037	0.006 (0.251)	0.03	6.7	38.26
GM Myo F I1	16.34 ± 2.22	16.67 ± 2.30	0.884 (0.82–0.93)	0.204	0.567	0.329 (0.126)	0.849	4.89	17.12
GM Myo F I2	16.60 ± 2.26	16.57 ± 2.27	0.832 (0.75–0.89)	0.267	0.741	0.038 (0.804)	0.922	5.5	28.41
GM Myo S I1	286.69 ± 54.85	291.22 ± 54.52	0.859 (0.79–0.91)	5.365	14.872	4.527 (0.180)	20.207	6.7	27.76
GM Myo S I2	294.20 ± 55.45	295.37 ± 59.75	0.788 (0.68–0.86)	8.131	22.537	1.173 (0.788)	24.97	8.04	43.62
GM Myo D I1	1.04 ± 0.15	1.03 ± 0.13	0.637 (0.48–0.75)	0.035	0.098	0.017 (0.228)	0.083	8.09	45.02
GM Myo D I2	1.05 ± 0.17	1.03 ± 0.14	0.596 (0.43–0.73)	0.041	0.113	0.026 (0.112)	0.091	8.83	50.78
GM Myo R I1	18.08 ± 2.84	17.83 ± 2.92	0.867 (0.80–0.91)	0.296	0.820	0.250 (0.150)	1.147	6.81	42.27
GM Myo R I2	17.76 ± 2.80	17.82 ± 2.85	0.850 (0.77–0.90)	0.315	0.873	0.055 (0.759)	1.15	6.79	30.19
GM Myo C I1	1.10 ± 0.16	1.08 ± 0.16	0.808 (0.71–0.87)	0.024	0.066	0.017 (0.157)	0.076	7.34	40.15
GM Myo C I2	1.08 ± 0.16	1.09 ± 0.16	0.831 (0.75–0.89)	0.020	0.055	0.003 (0.761)	0.068	6.6	28.57
ktW norm I1	13.10 ± 3.20	13.19 ± 3.39	0.969 (0.95–0.98)	0.077	0.213	0.092 (0.335)	0.619	5.25	25.93
ktW norm I2	13.08 ± 3.36	13.14 ± 3.46	0.948 (0.92–0.97)	0.124	0.344	0.057 (0.660)	0.77	6.51	34.62
ktW device I1	12.40 ± 3.57	12.28 ± 3.63	0.940 (0.91–0.96)	0.144	0.400	0.122 (0.399)	0.834	7.75	54.19
ktW device I2	12.30 ± 3.58	12.49 ± 3.46	0.942 (0.91–0.96)	0.158	0.439	0.191 (0.171)	0.931	8.19	28.08

MTh = muscle thickness; ST = stiffness; GM = gastrocnemius medialis (MTh GM and ST GM were measured with ultrasound SWE); AT = Achilles tendon; Myo = Myoton; (Myoton parameter: *F* = (oscillation) frequency, *S* = stiffness, *D* = elasticity, *R* = relaxation, *C* = creep); ktW norm = Standard knee-to-wall test; ktW device = Knee-to-wall test with device; I1 = Investigator 1; I2 = Investigator 2

Objectivity (investigators 1 and 2, both testing days).

For ultrasound muscle thickness and ST evaluation, there were high-to-excellent interrater reliability classifications in adherence to Koo & Li (2016) with ICCs of 0.87–0.91 for muscle thickness and 0.61–0.79 for SWE (moderate-to-high ICCs). The random error for objectivity was reported with 5.09% and 7.19% for muscle thickness and 13.17% and 14.85% MAPE for SWE. Myotonometry showed moderate-to-high relative reliability with ICCs ranging from 0.76 to 0.87 for Achilles tendon parameters and 0.66–0.97 for muscle parameters. Only for one parameter (GM Myo R T1), the systematic bias surpassed the MDC. Again, the random error peaked for the decrement parameters at the AT with MAPE > 10% (Table 4).

**Table 4:** Detailed objectivity statistics, including descriptives (*M* ± SD), the standard error of measurement (SEM), the minimal detectable change (MDC), as well as the systematic bias with inference statistics, while the mean absolute error (MAE), the mean absolute percentage error (MAPE), and the maximal percentage error (MPE) were used to quantify secondary variance

Parameter	I1	I2	ICC (95% CI)	SEM	MDC	Systematic bias	MAE	MAPE (%)	MPE (%)
MTh GM T1	1.90 ± 0.31	1.85 ± 0.30	0.905 (0.85–0.94)	0.020	0.055	0.044 (0.003)	0.09	5.09	29.68
MTh GM T2	1.87 ± 0.32	1.83 ± 0.31	0.868 (0.80–0.92)	0.032	0.089	0.043 (0.020)	0.125	7.19	27.74
ST GM T1	10.94 ± 2.82	11.26 ± 2.60	0.788 (0.68–0.86)	0.441	1.223	0.315 (0.123)	1.355	13.17	59.53
ST GM T2	10.94 ± 2.90	10.45 ± 2.13	0.613 (0.45–0.74)	0.667	1.849	0.495 (0.056)	1.517	14.85	96.43
AT Myo F T1	31.17 ± 2.60	30.92 ± 2.67	0.849 (0.77–0.90)	0.331	0.917	0.247 (0.142)	1.204	3.91	12.20
AT Myo F T2	30.58 ± 2.53	30.50 ± 2.73	0.875 (0.81–0.92)	0.247	0.684	0.082 (0.592)	0.987	3.3	17.6
AT Myo S T1	783.45 ± 95.81	795.28 ± 100.07	0.841 (0.76–0.90)	10.607	29.402	11.833 (0.063)	37.62	4.94	47.67
AT Myo S T2	776.01 ± 80.04	781.58 ± 92.33	0.831 (0.75–0.89)	11.286	31.285	5.573 (0.339)	38.827	5.07	21.85
AT Myo D T1	0.89 ± 0.19	0.89 ± 0.19	0.795 (0.69–0.87)	0.030	0.084	0.002 (0.883)	0.094	10.63	36.59
AT Myo D T2	0.89 ± 0.195	0.90 ± 0.18	0.758 (0.64–0.84)	0.033	0.091	0.010 (0.498)	0.095	10.52	39
AT Myo R T1	6.50 ± 0.84	6.35 ± 0.82	0.883 (0.82–0.92)	0.071	0.196	0.142 (0.002)	0.293	4.7	21.9
AT Myo R T2	6.52 ± 0.74	6.43 ± 0.91	0.786 (0.68–0.86)	0.102	0.284	0.056 (0.251)	0.031	4.85	20.54
AT Myo C T1	0.43 ± 0.05	0.43 ± 0.05	0.823 (0.73–0.88)	0.005	0.015	0.009 (0.005)	0.018	4.31	45.45
AT Myo C T2	0.44 ± 0.04	0.43 ± 0.05	0.865 (0.80–0.91)	0.005	0.013	0.004 (0.121)	0.018	4.08	17.11
GM Myo F T1	16.34 ± 2.22	16.60 ± 2.26	0.860 (0.79–0.91)	0.160	0.444	0.263 (0.054)	0.606	3.5	28.76
GM Myo F T2	16.67 ± 2.30	16.57 ± 2.27	0.957 (0.93–0.97)	0.072	0.200	0.103 (0.185)	0.491	3	17.42
GM Myo S T1	286.69 ± 54.85	294.20 ± 55.45	0.904 (0.85—0.94)	3.015	8.356	7.507 (0.007)	13.76	4.39	29.7
GM Myo S T2	291.22 ± 54.52	295.37 ± 59.75	0.939 (0.91–0.96)	2.027	5.619	4.153 (0.072)	11.607	3.74	24.62
GM Myo D T1	1.04 ± 0.15	1.05 ± 0.17	0.661 (0.51–0.77)	0.030	0.084	0.010 (0.496)	0.074	6.79	57.73
GM Myo D T2	1.03 ± 0.13	1.03 ± 0.14	0.891 (0.83–0.93)	0.012	0.033	0.001 (0.875)	0.051	4.89	16.56
GM Myo R T1	18.08 ± 2.84	17.76 ± 2.80	0.937 (0.90–0.96)	0.111	0.306	0.316 (0.005)	0.623	3.82	33.6
GM Myo R T2	17.83 ± 2.92	17.82 ± 2.85	0.973 (0.96–0.98)	0.063	0.174	0.011 (0.891)	0.54	3.05	8.06
GM Myo C T1	1.10 ± 0.16	1.08 ± 0.16	0.883 (0.82–0.92)	0.010	0.028	0.019 (0.033)	0.042	4.15	41.63
GM Myo C T2	1.08 ± 0.16	1.09 ± 0.16	0.970 (0.95–0.98)	0.004	0.10	0.001 (0.892)	0.03	2.79	9.38
KtW Norm T1	13.10 ± 3.20	13.08 ± 3.36	0.968 (0.95–0.98)	0.073	0.202	0.017 (0.864)	0.577	4.91	33.34
ktW norm T2	13.19 ± 3.39	13.14 ± 3.46	0.986 (0.98–0.99)	0.038	0.106	0.052 (0.437)	0.459	3.73	12.5
ktW device T1	12.40 ± 3.57	12.30 ± 3.58	0.966 (0.95–0.98)	0.092	0.255	0.106 (0.331)	0.705	6.44	25.77
ktW device T2	12.28 ± 3.63	12.49 ± 3.46	0.957 (0.93–0.97)	0.117	0.323	0.207 (0.085)	0.795	7.24	27.38

MTh = muscle thickness; ST = stiffness; GM = gastrocnemius medialis (MTh GM and ST GM were measured with ultrasound SWE); AT = Achilles tendon; Myo = Myoton; (Myoton parameter: *F* = (oscillation) frequency, *S* = stiffness, *D* = elasticity, *R* = relaxation, *C* = creep); ktW Norm = Standard knee-to-wall test; ktW device = Knee-to-wall test with device; I1 = Investigator 1; I2 = Investigator 2; T1 = day 1; T2 = day 2

### Agreement between ST measured via myotonometry and shear-wave elastography

ST determinations performed via MyotonPRO and SWE were *z*-transformed for agreement analysis, which prohibited a systematic bias. ICCs showed no significant relative agreement (ICC = 0.00–0.09), while LoAs ranged from − 2.81 to 2.81. Due to *z* transformation, no systematic error could occur.

### Agreement between KtW with and without measurement device

While the ICCs for agreement showed high-to-excellent reliability (ICC = 0.94, 0.90–0.96, 0.93, 0.89–0.95, 0.94, 0.92–0.97, and 0.96, 0.94–0.98), with means of 13.08–13.19 cm against 12.28–12.49 cm, there were significant systematic errors between the normal ktW and the ktW device (*p* < 0.001, *d* = 0.57–0.79). The MAE showed mean secondary variance between tests of 0.89–1.16 cm, which corresponds to 7.85–11.02%. Limits of agreement were for the first tester and first day: − 1.69–3.08 cm, for the second test and first day: − 1.81–3.38 cm, for the first tester on the second day: − 1.35–3.17 cm and for the second tester on the second day: − 1.27–2.57 cm.

### Influence of muscle and tendon ST on lower limb ROM measured via the KtW

**Fig. 4:**
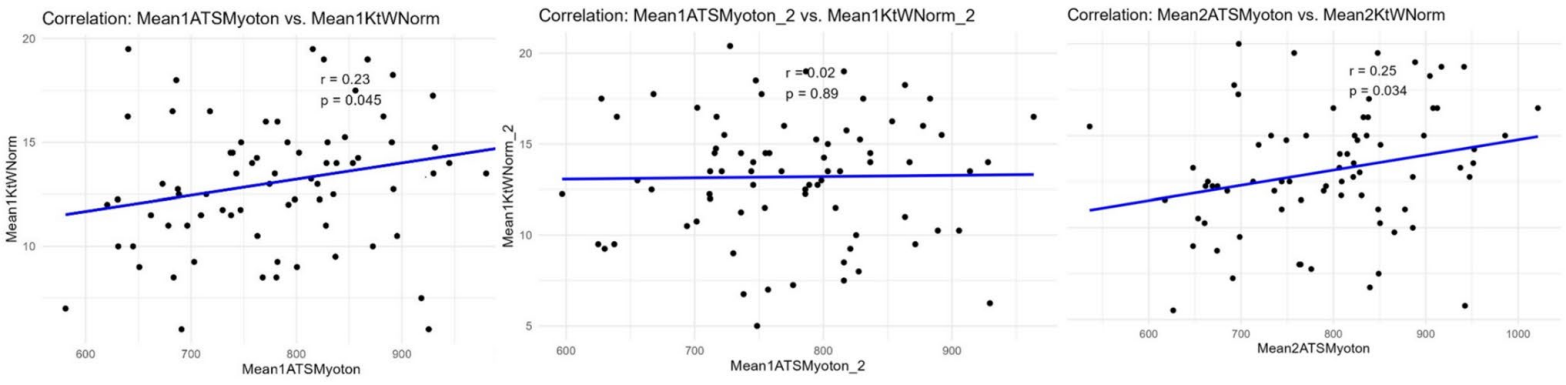
Illustrates correlation analyses between ST measured with different devices, on both days and between investigators

While there were small significant correlations in the first investigator for SWE ST on ROM (*r* = 0.21–0.26, *p* = 0.024–0.06), there were negative but trivial non-significant effects in the second investigator (*r* = − 0.024 – − 0.074, *p* = 0.53–0.84) when ROM was measured via the normal KtW. When using the KtW device, the first investigator had non-significant-to-significant positive correlations with SWE ST (*r* = 0.19–0.30, *p* = 0.01–0.17), while for the second investigator, there were non-significant but positive and negative relationships (*r* = − 0.05–0.02). In contrast, for myotonometry to the normal KtW, there was no significant relationship to muscle ST (*p* = 0.25–0.75) as well as when measured with the KtW device (*p* = 0.47–0.90) (see Fig. 4).

## Discussion

With this study, we sought to investigate the origin of errors of repeated measures to investigate whether differences between measurement days or investigators relevantly affected the correlation between muscle- and tendon stiffness on ROM. In accordance with the literature, we found excellent and satisfying intraday reliability with, overall, no systematic errors. Secondary variance for intraday stayed below 5%, while both myotonometry and SWE showed clinically relevant deficits in interday repeatability and interrater reliability/objectivity. These deficits cause a meaningful bias which diminishes the validity of ST evaluation methods, observable in a relationship dependency on investigator and day. We saw that, while in the first investigator, there was a small relationship between ST and ROM, when the same ST values of the second investigator were evaluated, the significant correlation disappeared. This may be due to significant standardization problems related to commonly performed measurement protocols or the fact that we are completely unaware of which ST determination methods are valid: While both ST measurements were individually conducted under reliable conditions, there was literally no agreement between SWE and myotonometry. Therefore, the results in this study call previous ST evaluations and subsequent clinical recommendations into question.

### Ultrasound investigations

Literature substantially lacks detailed reliability exploration studies for ultrasound, especially when performing SWE and cross-sectional study designs. Study quality limitations were outlined in a recent review including 17 reliability studies in the older participants (Nijholt et al. 2017) *“Despite high scores on methodological quality, we found that information regarding the scanning procedure was unclear in most of the reliability studies. In particular, information was lacking on the scanning position and marking of the skin.” (p.710)* while relative intraday reliability was excellent (majorly) across the literature (ICC ≥ 0.9) (Leong et al. 2013; Šarabon et al. 2019; Taş et al. 2017). These, as well as reported interday reliability data, are in accordance with relative reliability observed in the presented study, as in our study, interday quantifications showed reduced ICCs over 0.81–0.91 (Taş et al. 2017) to 0.34–0.45 (Sarabon et al. 2019). Also, related imaging techniques such as supersonic shear imaging resulted in interday reliability of ICC = 0.82 (Lacourpaille et al. 2012). Although further examples exist in the literature (Brandenburg et al. 2015; Dubois et al. 2015; Phan et al. 2019), the large discrepancy in evidence underlines that there seems to be a large variance in standardization, resulting in investigation-specific ICCs. Also, the range of ICCs covered indicate that, while within one session, assessors might be able to reproduce their own results when testing several times in a row (good-to-excellent intraday reliability (Phan et al. 2019), neither between the days nor between investigators results can be reproduced consistently. Relative inter-investigator reliability/objectivity found in this study is, in parts, confirmed by previous results as well: Leong et al. (2013) showed upper trapezius inter-investigator reliability of ICC = 0.78–0.83 in 28 healthy adults and classified those as excellent. Šarabon et al. (2019) also stated inter-rater reproducibility of ICC = 0.74–0.88 (depending on performed with a machine or by a human), while Lacourpaille et al. (2012) reached ICCs of 0.71. Although the literature reported these values, overall, as satisfying, we cannot agree with this general classification.

This critical perspective stems from several articles around the new millennium outlining the relevance of detailed measurement error statistics in reliability and objectivity studies that were categorically neglected in the current literature. Current authors can follow an extensive discussion, for instance, provided by Hopkins (2000), Atkinson and Nevill (2000, 1998), or Barnhart et al. (2007), who highlighted the relevance of conducting reliable measurement protocols. They explicitly referred to lacking validity of exclusively reporting relative reliability indices, as those did not account for systematic, as well as random measurement errors (Hopkins 2000). Therefore, as early as more than 20 years ago, there is a urgent need to account for those measurement problems arising from lacking standardization or systematically occurring problems in data collections, such as learning effects in unfamiliar testing conditions (Warneke et al. 2023a, b, c), or randomly upcoming divergences in evaluation protocols. For ultrasound imaging in general, these were outlined to be subjective factors mostly attributable to the investigator such as changing pressure, as well as rotation and angle displacements of the probe (Kristijansson et al. 2004; Šarabon et al. 2019; Warneke et al. 2022a, b). This problem was partly solved in Šarabon et al. (2019) who used a device to standardize the protocol. However, they also showed that there was extreme sensitivity, underlining our request for improved standardization protocols here: The authors reported *“In conclusion, SWE is a reliable tool for assessing muscle ST if the muscle is examined in relaxed condition, while changing the force applied with the probe for as little as 1.5 N results in significantly lower repeatability.”*

Although some individual articles sought to account for absolute measurement errors and practical interpretability by providing the SEM and the MDC (Leong et al. 2013; Zhou et al. 2024), SEM/MDC are calculated using the ICC, thus being limited advantageous. Hopkins, in contrast, suggested the implementation of the typical error (TE), while Atkinson and Nevill preferred providing BA analyses with focus on the LoAs. However, we were unable to find SWE studies performing these analyses. Therefore, our study was the first that accounted for these error sources by providing the systematic error as well as the random error and quantified the random error via the MAE and MAPE, which can be considered a quantification of qualitative analyzing the BA plot. When reviewing the random error that accompanied the ICCs that were classified as satisfying, alone in intraday reliability, we found an MAPE of up to 5%. Interday secondary variance reached a mean error of ≥ 20% with MPE of > 80%. These measurement errors must be considered when interpreting results in, for example, Young's moduli in the plantar flexors and AT after the 4-week stretching program of 12.2 kPa and 40.1 kPa, respectively (Cummings et al. 2022; Miyasaka et al. 2024) Accordingly, we cannot confirm sufficient reliability in SWE determinations in general, as it cannot be ruled out that reported ICCs (which are very ambivalent across the literature) were accompanied with clinically relevant measurement errors. To anticipate, measurement errors are considered clinically relevant when impacting clinical and practical recommendations stemming from this data collection or affect further analysis results (see section below).

### Myotonometry

Easily applicable and independent ST measures were promised by the MyotonPRO device (Myoton AS, Tallin, Estonia). While the literature also provides us with several articles highlighting sufficient reliability to collect practically relevant data (0.80–0.93, and 0.4 for the rectus femoris (Bizzini and Mannion 2003; Agoriwo et al. 2022), the wide range of indices from SWE is available for myotonometry as well: Lee et al. 2021 indicated ICCs for intrarater reliability of 0.94, however with 95% CI 0.15–0.99 for the rectus femoris or in the biceps femoris even with ICC = 0.88, − 0.38–0.98. Although the mean ICC suggests reasonable reliability, one cannot neglect the 95% CI from a statistical perspective. This problem can also be reviewed in a recent article published on the MyotonPRO evaluation for skin and muscle showing between day reliability of ICC = 0.77–0.91 for skin and 0.49–0.99 for the muscle probe. Interestingly, for the Myoton muscle probe, Table 3 of John et al. (2023) in the study provided data for the right leg of 0.17 (− 1.38–0.72) and for the right forearm of 0.48 (− 0.42–0.82), not prohibiting the classification of the MyotonPRO as a reliable tool: *“The MyotonPRO showed good intra‐ and inter‐rater reliability for the ST for both muscle and skin Probe.”* (John et al. 2023). Also, for MyotonPRO assessments, in accordance with our study showing intraday reliability classified as excellent, the relative reliability dropped when evaluating the interday reliability (ICC ≥ 0.7) (Aird et al. 2012; Bravo-Sánchez et al. 2022). When reviewing results provided in the literature, we obtained superior reliability as our ICCs were partly even better. Nevertheless, interday reliability still reached MAPEs with up to 9% (which is below those of the SWE investigation). Since no previous study performed an accurate measurement error evaluation, the provided systematic and random error evaluation is the first in literature showing actual measurement errors, which accompany relative reliability and objectivity indices.

### Range of motion assessment

The KtW is a frequently performed measurement to assess “functional ROM” in the lower limb, often applied in clinical settings, e.g., injury prediction (Bowen et al. 2019; Harris et al. 2022; Silva et al. 2018). To improve precision and sensitivity, Warneke et al. (2022a, b, 2023a, b, c) introduced a KtW device, which was stated to provide excellent reliability (ICC: 0.98–0.99). However, also these articles did not account for interday reliability, while also neglecting associated systematic and random measurement errors. Thus, the device can be considered not validated. This is the first study that opposed device-based ROM assessment to the commonly performed KtW (Warneke et al. 2023a, b, c, 2024). While both testing types showed excellent relative reliability, the MAPE of the device was in all cases higher compared to the common test. This leads to a systematic error between the tests and suggests lower performance values when measured with the device. While on the first view, measurement values do not support the use of the KtW device, this is a well-fitting example to discuss the inverse relationship of testing precision/sensitivity and reliability. Obviously, the device can detect performance with a precision of 1 mm, while the ktW was performed with 0.5 cm accuracy. Therefore, if a participant performed 10 or 10.4 cm, with the common KtW, this would result in 10 cm in both cases (100% agreement), as the 10.5 cm were not tested/could not be evaluated. In contrast, measured with the device, there was a discrepancy of 4%, which could account for higher measurement errors in absolute stemming from improved accuracy of the device.

### Clinical relevance and practical applications

Problems in reliability (inter- and intraday) as well as objectivity have relevant implications for derived recommendations, especially in cross-sectional studies in which no control group is available. As outlined in the introduction, there are contrasting results on the influence of ST on ROM, which could arise from reliability, validity, and objectivity problems of the ST evaluations. While ST evaluated by the first investigator significantly impacted ROM to a small magnitude, no such effects were found when another investigator performed the ST measurements. This problem must be considered relevant as intraday reliability (the most frequently reported reliability metrics, if any exists Aird et al. 2012; Betz et al. 2021; Bizzini and Mannion 2003; Lee et al. 2021), was excellent, interday and objectivity were classified sufficient (relative reliability in accordance with the literature). However, also the device with which the ST was determined played a role. There was literally no agreement between ST evaluations performed with MyotonPRO and SWE, indicating the evaluation of two separated parameters. Of note, Lee et al. (2021) sought to determine the agreement between both devices. Unfortunately, again, inappropriate statistical procedures bias the results: Correlation coefficients of *r* = 0.42–0.67 do not provide any statement about agreement (Lin 1989). Therefore, to derive clinically relevant statements from ST evaluations, first, there is an urgent need for a clear definition of ST and how it is to evaluate (see difference between Young’s modulus and shear modulus, for example). Second, measurement error quantification underlines the importance of well-performed standardization protocols when working with such sensitive and precise devices such as ultrasound sonography in general, and SWE in special (Šarabon et al. 2019; Warneke et al. 2022a, b). Before measurement problems are not solved, results of studies performing ST determinations must be reviewed very carefully.

### Limitations

While a G-Power analysis is considered standard and a requirement of several scientific Journals, the validity must be questioned especially for correlation analysis. A content-based sample size inclusion is required, especially since Schoenbroedt and Perugini (2013) outlined that correlations only stabilize in large samples, which is also true for ICCs (Warneke et al. 2025). Although we recruited a much larger sample size than most other studies, a doubling of the sample size to reach stable correlation coefficients would be required. Therefore, still, it cannot be ruled out that the results are statistically underpowered. In addition, while we adhered to a protocol for our ultrasound measurements, the general challenge of standardizing such measurements remains. Variables such as the angle of the ultrasound probe, the applied pressure, and the experience of the examiner can all influence the results (Warneke et al. 2025a, b, c). Although it seems questionable whether this is a limitation of this specific study or an overall problem in measurement, there is an urgent need for establishing internationally guidelines and standardization protocols to minimize interday- but especially interinvestigator affectors and their relevance on result interpretation.

Furthermore, tendon ST was only evaluated via myotonometry due to limited measurement possibilities in the specific SWE device (Ryu and Jeong 2017). Therefore, the correlations outlined here were only evaluated for the gastrocnemius, not for the Achilles tendon, calling for future studies to evaluate the relationship, but also reliability differences between myotonometry and SWE in tendons (i.e., the Achilles tendon). Furthermore, as ROM was tested via the KtW, depending on the available ankle ROM, the knee and hip joint are involved to the movement which might affect the ROM test results. Since the test can be considered kind of a coordinative task, it must be seen as a limitation that no previous habituation session for this test was performed. However, still, we saw comparatively small measurement errors and excellent ICCs in this evaluation.

Another limitation is that tissue stiffness might be joint-specific and depending on investigating active or passive stiffness. While the ankle joint has small degrees of freedom compared to, for example, the shoulder or the hip joint, this aspect could affect the correlation between muscle- and tendon stiffness and ROM. Furthermore, in the current study, only passive stiffness values were evaluated, while active stiffness assessments are highly warranted. This should kept in mind in designing future studies that could explore the transferability of the found results to other muscles and tendons.

## Data Availability

Original data can be requested from the corresponding author upon reasonable request.

## Abbreviations

ICC: Interclass correlation coefficient
KtW: Knee-to-wall test
MDC: Minimal detectable change
ROM: Range of motion
SEM: Standard error of measurement
ST: Stiffness
SWE: Shear-wave elastography
